# Microbiome of the Proximal Small Intestine in Patients with Acute Pancreatitis

**DOI:** 10.3390/diagnostics15151911

**Published:** 2025-07-30

**Authors:** Vladimir V. Kiselev, Stanislav I. Koshechkin, Alexey V. Kurenkov, Vera E. Odintsova, Maria S. Zhigalova, Alekxandr V. Tyakht, Sergey S. Petrikov, Petr A. Yartsev, Ilya V. Dmitriev

**Affiliations:** 1 Sklifosovsky Research Institute for Emergency Medicine, 3 Bolshaya Sukharevskaya Square, 129090 Moscow, Russia; kiselevvv@sklif.mos.ru (V.V.K.); oky-doker@yandex.ru (A.V.K.); zhigalovams@sklif.mos.ru (M.S.Z.); petrikovss@sklif.mos.ru (S.S.P.); yartsevpa@sklif.mos.ru (P.A.Y.); 2 Nobias Technologies LLC, 14B Berezovaya Alleya St., Bldg. 2, Prem. 2/4, 127273 Moscow, Russia; st.koshechkin@gmail.com (S.I.K.); vera.odints@gmail.com (V.E.O.); 3 FSBIS Institute of Gene Biology, Russian Academy of Sciences, 34/5 Vavilova St., 119334 Moscow, Russia; a.tyakht@gmail.com; 4 Scientific and Educational Institute of Continuing Professional Education Named After N.D. Yushchuk, Federal State Budgetary Educational Institution of Higher Education, “Russian University of Medicine” of the Ministry of Health of the Russian Federation, 4 Dolgorukovskaya St., 127006 Moscow, Russia; 5 Federal State Autonomous Educational Institution of Higher Education, Pirogov Russian National Research Medical University, 1/6 Ostrovitianova St., 117513 Moscow, Russia

**Keywords:** acute pancreatitis, severity, severity scales, small intestine microbiome, microbial pancreatitis severity index

## Abstract

Currently, due to the complexity of obtaining samples, specific features of laboratory processing and analysis of the results, there is a lack of data on the microbial signature of the small intestine in healthy and diseased states of the upper gastrointestinal tract. **Objective:** To investigate the characteristics of the small intestinal microbiome in acute pancreatitis of varying severity and to identify correlations with clinical factors. **Methods:** This study included 30 patients with acute pancreatitis of varying severity treated between 1 January 2019 and 31 December 2021. The composition of the microbiota was analyzed by metagenomic sequencing of the 16S rRNA gene from jejunal samples. **Results:** The mortality rate in the study group was 23.3%. The small intestinal microbiome was dominated by *Streptococcus* (median relative abundance 19.2%, interquartile range 6.4–35.1%), *Veillonella* (3.4%; 0.6–7%), *Granulicatella* (2.7%; 0.6–5%), *Fusobacterium* (2.2%; 0.3–5.9%), *Prevotella* (1.5%; 0.3–8%), *Haemophilus* (0.9%; 0.2–10%), *Gemella* (0.8%; 0.2–4.3%), and *Lactobacillus* (0.2%; 0.1–0.9%). More severe disease was associated with decreased abundance of *Neisseria mucosa, Parvimonas micra,* and *Megasphaera micronuciformis*. In contrast, the relative abundance of the genera *Streptococcus* (species *S. rubneri*/*parasanguinis*/*australis*), Actinomyces, and several genera within the family Enterobacteriaceae was higher in these patients. **Conclusions:** The state of the microbiota has important prognostic value and correlates with the duration from the onset of the pain syndrome to the time of receiving qualified care in the hospital.

## 1. Introduction

Acute pancreatitis (AP) is characterized by inflammation of the exocrine part of the pancreas and is associated with damage to acinar cells and with the development of a local and systemic inflammatory reaction. AP can range in severity from edema, often characterized by spontaneous resolution, to severe systemic inflammation with pancreatic tissue necrosis, multi-organ failure, and death [1,2]. Currently, AP is one of the most common causes of hospitalization among all gastrointestinal diseases. The global incidence of AP is 13–45 cases per 100,000 adult population per year [3]. There is an increase in the incidence of AP in both North America and Europe; today, it is 340 cases per million people per year [3]. Over the past decade, the incidence of AP has tended to increase steadily in most countries [3,4]. Among acute abdominal diseases, AP ranks first (24.1%), ahead of acute appendicitis (23.4%) and acute cholecystitis (23.8%) [4].

In current systems, scales, and classifications of AP, there is virtually no factor or criterion that reflects the extent of damage to the intestinal wall and the state of the intestine as a whole. Data on the important role of the intestine and its microbiota in the development of AP complications have been available for many years [5,6,7]. There has been a large number of reports over the last decade on the significant role of the intestinal microbiota in the development of AP and its effect on the severity and outcome of the disease [8,9,10,11,12,13]. These findings highlight the importance of intestinal homeostasis disruptions, which can contribute to disease progression in AP and potentially trigger organ dysfunction and sepsis [14,15,16,17].

Despite extensive literature from both clinical and experimental studies characterizing the human microbiome, data on the microbial signature of the small intestine remain insufficient due to the complexity of sample collection, specific laboratory processing requirements, and challenges in data analysis [18]. Most of the studies focused mainly on the analysis of the microbial composition of feces, because feces sampling is a non-invasive procedure and allows for the composition of the colon microbiota to be assessed [19,20]. However, due to the significantly higher concentration of bacteria in the lower gastrointestinal tract, fecal samples cannot provide reliable information about the composition of the small intestine. At the same time, investigating the small intestinal microbiome is a fundamentally important task, as its intraluminal environment differs markedly from other parts of the gastrointestinal tract. This is due to its vast surface area, active peristalsis, oxygen-rich environment, the presence of pancreatic and bile secretions, antimicrobial peptides produced by Paneth cells, brush border digestion, and the absorption of the majority of nutrients [21,22,23,24]—all of which directly affect intestinal function. Moreover, given the localization of pathological processes in AP, the microbiome of the small intestine may be involved in influencing the severity of the disease and the further development of septic complications.

Clearly, studying the small intestinal microbiome is critical for a deeper understanding of both normal and pathological processes in the upper gastrointestinal tract. The purpose of this study was to investigate the specific features of the small intestinal microbiome in patients with AP of varying severity and to identify correlations with clinical factors. To our knowledge, similar studies have not been previously reported in the literature, likely due to the methodological challenges of obtaining necessary biosamples from the small intestine, recruiting critically ill patients, and coordinating the complex laboratory and bioinformatic procedures required for source data acquisition and analysis.

## 2. Materials and Methods

### 2.1. Study Design

This cross-sectional, prospective, single-center, observational case-control study included patients diagnosed with acute pancreatitis (AP) who received treatment at the State Budgetary Healthcare Institution “Sklifosovsky Research Institute for Emergency Medicine” of the Moscow Health Department between 1 January 2019 and 31 December 2021. The study included patients admitted to the intensive care unit (ICU) with a referral diagnosis of acute pancreatitis (AP). The diagnosis was established based on the Revised Atlanta Classification (RAC) criteria for assessing the severity of AP [25], in combination with a typical clinical presentation. All patients received treatment in accordance with the IAP/APA guidelines for the management of AP [26].

#### 2.1.1. Inclusion Criteria

1. Confirmed diagnosis of AP;
2. Age 18–80 years;
3. Signed written informed consent to participate in the study.

#### 2.1.2. Exclusion Criteria (at Enrollment)

1. Antibacterial therapy within the two months preceding the AP episode;
2. Rehospitalization within six months following any prior inpatient treatment;
3. Presence of inflammatory diseases of the gastrointestinal tract;
4. HIV infection or other immunodeficiency conditions;
5. Malignancies of the gastrointestinal tract;
6. Atopic bronchial asthma or atopic dermatitis.

#### 2.1.3. Withdrawal Criteria

1. Withdrawal from treatment;
2. Patient’s refusal to participate at any stage of the study;
3. Violation of the procedure for obtaining biomaterial samples;
4. Violation of the biomaterial processing or biobanking procedures;
5. Failure of biomaterial samples to meet DNA quality control standards.

This study was approved by the ethics committee of the State Budgetary Healthcare Institution “Sklifosovsky Research Institute for Emergency Medicine” of the Moscow Health Department (Protocol No.8-20 dated 6 May 2020). This study was conducted in accordance with the ethical principles of the Declaration of Helsinki [27].

To assess disease severity, seven scoring systems were utilized: Ranson, SOFA, APACHE II, SAPS II, MARSHALL, BISAP, and Shock Index (SI). In addition, four clinical outcome factors were considered for case interpretation: mortality, surgical intervention, pneumonia, and severity classification according to the MAP/MSAP/SAP criteria. Based on these data, patients were stratified by disease severity and clinical prognosis. Data collection for scoring was carried out on the day of obtaining biomaterial samples (±12–20 h), ensuring that the assessments reflected the patient’s actual clinical status at the time of jejunal content sampling.

### 2.2. Microbiome Analysis

Biomaterial samples were taken during routine esophagogastrointestinoscopy using a lavage technique designed for sampling biological fluid from the small intestine (patent RU 2,738,007 C1: URL https://worldwide.espacenet.com/patent/search/family/073792432/publication/RU2738007C1?q=pn%3DRU2738007C1, (accessed on 24 July 2025)), with the aid of a multifunctional intestinal catheter (patent RU 199,398 U1: URL https://worldwide.espacenet.com/patent/search/family/072421196/publication/RU199398U1?q=pn%3DRU199398U1, (accessed on 24 July 2025)). The catheter was constructed specifically to exclude contamination of samples with microbiome of upper parts of gastrointestinal tract; the details may be found in the patent. The procedure was completed on the day of the patient’s admission to the intensive care unit, before the initiation of antibacterial and antisecretory therapy and before the initiation of enteral or perioral nutrition. Samples were transferred to a cryochamber at −39 °C within 5 min of collection and stored there for no longer than 7 days. They were then transported in a cryocontainer with a temperature control system and coolant to a long-term storage cryochamber at −80 °C until laboratory analysis began. Seven samples have already been described in [28].

Laboratory processing of samples was carried out according to the following protocol: DNA was extracted from small intestinal lavage and biopsy samples using the Qiagen Power Fecal PRO kit. Amplification of the V4 region of the 16S rRNA gene was carried out using the modified 515F primer (5′-GTGBCAGCMGCCGCGGTAA-3′) [19] and Pro-mod-805R (5′-GACTACNVGGGTMTCTAATCC-3′) [20]. A second round of amplification was performed using standard Illumina indexing primers with adapters. Both rounds of PCR were performed using PCR buffer (Eurogen, Moscow, Russia) and a Bio-Rad CFX-96 thermal cycler (Bio-Rad, Hercules, CA, USA). PCR products were purified using a DNA Cleanup Mini kit (Eurogen, Moscow, Russia). DNA concentration was measured using a Qubit fluorimeter (Invitrogen, Waltham, MA, USA) with the Quant-iT dsDNA High-Sensitivity Assay Kit. Purified amplicons were pooled equimolarly according to measured concentrations. Library preparation and sequencing were carried out using the MiSeq Reagent Kit v2 (500 cycles) and the MiSeq Sequencing System (Illumina, San Diego, CA, USA), following the manufacturer’s protocol. Initial processing has been described in detail in previous studies [20,29]. Paired-end reads were quality-checked and trimmed and then merged using SeqPrep. The average merged read length was 252 base pairs.

Taxonomic profiling was performed using amplicon sequence variants (ASVs) generated via the DADA2 v1.18, BLAST from https://blast.ncbi.nlm.nih.gov (accessed on 24 July 2025) [30]. The analysis was conducted at the ASV level, allowing for high-resolution discrimination of unique sequences. Taxonomic classification of the resulting sequences was further refined using the BLAST algorithm (2020 year Update) against the NCBI non-redundant (nr) database. In taxonomic annotations, the prefix s__ indicates species, g__ indicates genus, f__ indicates family, and s__u denotes an unclassified species within a known genus and family (e.g., s__u(g_*Veillonella*) refers to an unclassified species within the genus *Veillonella*). Each ASV was assigned a unique identifier to distinguish between individual sequence variants belonging to the same species.

Alpha diversity was assessed using Shannon’s index after fivefold rarefaction to 3000 reads per sample, with results averaged across all iterations. Pairwise dissimilarity between samples was evaluated using the Aitchison distance calculated based on the proportional abundance of prevailing ASVs—defined as those present in more than half of the samples or constituting at least 10% in any single sample. To account for potential contamination during sample processing, taxonomic composition was compared to that of three negative control samples (sterile water). Taxa that were abundant in the negative control and rare in the samples of interest were excluded for further analysis. Several taxa found in negative control samples were retained, as they are well-documented constituents of the intestinal microbiota.

### 2.3. Statistical Analysis

Statistical analysis was performed using R software version 4.3.3 [31].

Associations of various factors and microbiota composition were assessed using the NearestBalance package [32]. This approach accounts for the compositional nature of microbiome data, recognizing the mathematical constraints inherent in working with relative abundances. Instead of analyzing the relative abundance of individual microbes separately, the method identifies groups of microbes whose relative ratios are most strongly associated with the factor under study (e.g., disease severity). The ratio—referred to as a **balance**—is calculated as the difference between the mean logarithm of abundances of microbes in one group and the mean logarithm of abundances in the other group, multiplied by a scaling coefficient that depends on the number of taxa in each group. The formula of the balance is as follows:

$$balance(pos,\;neg)\;=\;\sqrt{N_{pos}N_{neg}/(N_{pos}{+N}_{neg})}\;\ln(\mathrm{gm}(\mathrm{pos})/\mathrm{gm}(\mathrm{neg}))$$

where “pos” and “neg” are the two groups of taxa (“positively” and “negatively” associated with a factor), $N_{pos}$ and $N_{neg}$ are the numbers of taxa in the groups, respectively, and gm denotes the geometrical mean of the taxa abundances. This balance-based approach provides a more accurate assessment of differences between samples [33,34]. The balance is found as an approximation of compositional shift associated with a unit change in the factor of interest, and the compositional shift is estimated as a coefficient of multivariate linear regression on the centered log-ratio transformed proportions.

The statistical significance of associations in beta diversity was assessed using the *adonis2* function from the *vegan* package, with Aitchison distance as the dissimilarity measure, given its suitability for evaluating differences in proportions of dominant taxa. Prior to analysis, zero values in the compositional dataset were replaced using the *cmultRepl* function from the zCompositions package in R [35].

To explore associations between clinical parameters, alpha diversity indices, and microbial balance values, Spearman’s rank correlation coefficient was used for comparisons between continuous variables, while the Mann–Whitney U test was applied for comparisons involving continuous and categorical variables.

The significance level in all statistical tests was 0.1. Multiple testing adjustment was performed using the Benjamini–Hochberg method.

## 3. Results

### 3.1. Cohort Description

According to the inclusion and exclusion criteria, 43 patients were initially enrolled in the study. Of these, 13 patients were subsequently withdrawn from the study cohort for the following reasons: withdrawal from treatment (*n* = 1), refusal to participate at specific study stages (*n* = 2), violation of the procedure of obtaining biomaterial samples (*n* = 1), violation of the biomaterial processing or biobanking procedures (*n* = 3), and failure to meet DNA quality control requirements (*n* = 6). In the latter cases, samples could not be analyzed due to insufficient bacterial DNA or the presence of PCR inhibitors.

Data from 30 patients were included in the further analysis. Male patients were significantly more prevalent, accounting for 76.7% of the cohort compared to 23.3% females (*p* = 0.015). The mean age of patients with AP was 47 (36; 54) years. A substantial proportion of patients fell within the 41–59 age group, which also showed the highest prevalence of moderate and severe forms of AP (Table 1).

The most common etiological factors of AP in the study cohort were alcohol-related (*n* = 22, 73.3%) and biliary (*n* = 5, 16.6%) (Table 2).

### 3.2. Time of Admission of Patients from the Onset of Pain Syndrome

The interval between the onset of abdominal pain and admission to the intensive care unit (ICU) ranged from 0 to 7 days. Twelve patients were admitted within the first 24 h, nine between 24 and 48 h, and nine more than 48 h after symptom onset.

### 3.3. Clinical Outcomes of Acute Pancreatitis

Eleven patients developed purulent-septic complications associated with infection of areas of pancreatogenic necrosis in the retroperitoneal fat tissue, which required minimally invasive surgical interventions combining percutaneous and transluminal staged treatment of local acute pancreatitis (AP) complications. Renal replacement therapy was performed in 18 patients due to acute renal injury. Seven deaths were recorded in patients with progressive multiple organ failure.

### 3.4. Severity Assessment

Severity characteristics based on the clinical scoring systems used are summarized in Table 3.

### 3.5. Microbiome Composition

**Figure 1:**
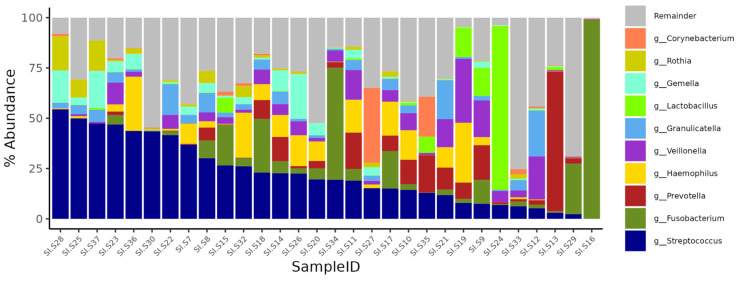
Composition of microbiota samples. Each bar represents an individual sample; colors indicate bacterial genera.

A total of 30 samples from 30 patients were analyzed. All samples were collected on the day of admission before starting before starting of antibacterial and antisecretory therapy and before initiation of enteral or perioral nutrition. The most abundant bacterial genera are presented in Figure 1. The small intestinal microbiome was predominantly composed of *Streptococcus* (median relative abundance 19.2%, interquartile range [IQR] 6.4–35.1%), *Veillonella* (3.4%; IQR 0.6–7%), *Granulicatella* (2.7%; IQR 0.6–5%), *Fusobacterium* (2.2%; IQR 0.3–5.9%), *Prevotella* (1.5%; IQR 0.3–8%), *Haemophilus* (0.9%; IQR 0.2–10%), *Gemella* (0.8%; IQR 0.2–4.3%), and *Lactobacillus* (0.2%; IQR 0.1–0.9%). These genera are commonly reported in studies of the oral microbiome, where *Streptococcus* and *Veillonella* may comprise up to 50% of the total bacterial biomass [36]. However, their presence in the large intestine has also been described [37]. The mean Shannon alpha diversity index was 3.8 ± 1.1.

Overall, the observed microbial composition is consistent with current knowledge of the human microbiome [9,38,39]. A more rigorous description of the dysbiosis in patients would require a control group of healthy subjects. We did not include a control group in our study because of ethical reasons; the sample collection is too invasive. This decision is typical for studies of the human small intestinal microbiome [40]. The existing studies differ in the sample collection methods. So, we focused on exploring variability in the patient samples and its association with phenotype rather than describing the dysbiosis in acute pancreatitis.

### 3.6. Associations Between Clinical Indicators and Microbiome

As illustrated in Figure 1, the microbial composition varied considerably across samples. Further analysis was aimed at determining whether this variability was associated with differences in clinical severity, namely, all scales listed in Table 3, MAP/MSAP/SAP classification, need for surgical intervention due to purulent-septic complications, pneumonia, and survival or death.

#### 3.6.1. Alpha and Beta Diversity

Based on the analysis of alpha and beta diversity, almost no strong association was observed between these metrics and overall microbial composition severity measures (p.adj > 0.1; see Table A1), with the exception of a potential association between Shannon alpha diversity and systemic inflammatory response syndrome (SIRS) (p.adj = 0.06) and potential association between Aitchison beta diversity and the SAPS II score (p.adj = 0.099) and the need for surgery (p.adj = 0.099). Shannon alpha diversity showed a moderate positive correlation with SIRS (Spearman’s ρ = 0.49), and SAPS II explained about 6% (R2 = 0.061) of beta-diversity variation, as well as the need for surgery (R2 = 0.063).

#### 3.6.2. Pancreatitis Severity Microbial Index (PSMI)

**Figure 2:**
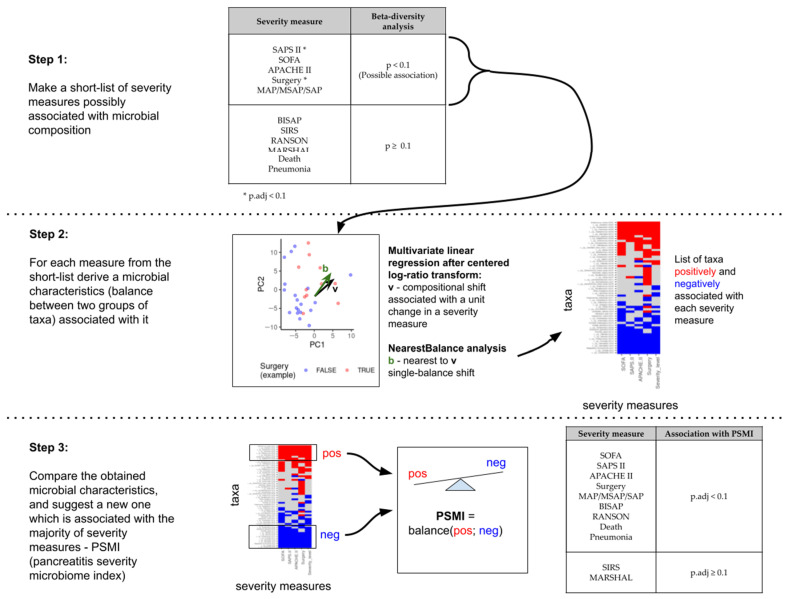
Schematic illustration of taxa proportions analysis and the definition of the pancreatitis severity microbial index (PSMI). MAP—mild acute pancreatitis, MSAP—moderately severe acute pancreatitis, SAP—severe acute pancreatitis.

Next, we analyzed taxa proportions looking for microbiome characteristics associated with the severity of acute pancreatitis in the most general sense, namely, correlated with various measures of severity. The workflow of the analysis is shown in Figure 2.

As the first step, we looked at the results of beta-diversity analysis and selected severity measured with uncorrected for multiple testing *p*-values < 0.1 (Table A1). These were three scale measures (SOFA, SAPS II, and APACHE), need for surgery and severity level (MAP/MSAP/SAP) classification, and need for surgical intervention due to purulent-septic complications.

As the second step, we looked for microbial markers of these features using center ed log-ratio transformation followed by multivariate linear regression analysis and the nearest balance approximation of the result (Figure 2, step 2). The choice of the method was motivated by the well-known problem of taxa proportion interdependency: due to the lack of absolute microbial concentrations and normalization factors, direct comparison of the relative abundance of a single taxon across samples may result in misleading conclusions [33]; thus, specific compositional methods should be used. The used approach is one of such methods [31], namely, a balance-based approach. This method identifies two groups of taxa whose relative proportions are most strongly associated with the clinical parameter of interest. For instance, if group A taxa are more prevalent in one condition and group B taxa in another, the resulting balance—calculated as the log-ratio between the geometric means of the two groups, adjusted for group size (see formula in Methods) —yields a single continuous value representing the microbial signature of a given sample. This allowed us to identify bacterial groups linked to the five selected severity measures and calculate their corresponding balances for each sample. Statistical analysis showed that these balance values significantly correlated with clinical severity (p.adj < 0.1, Table A2).

**Figure A1:**
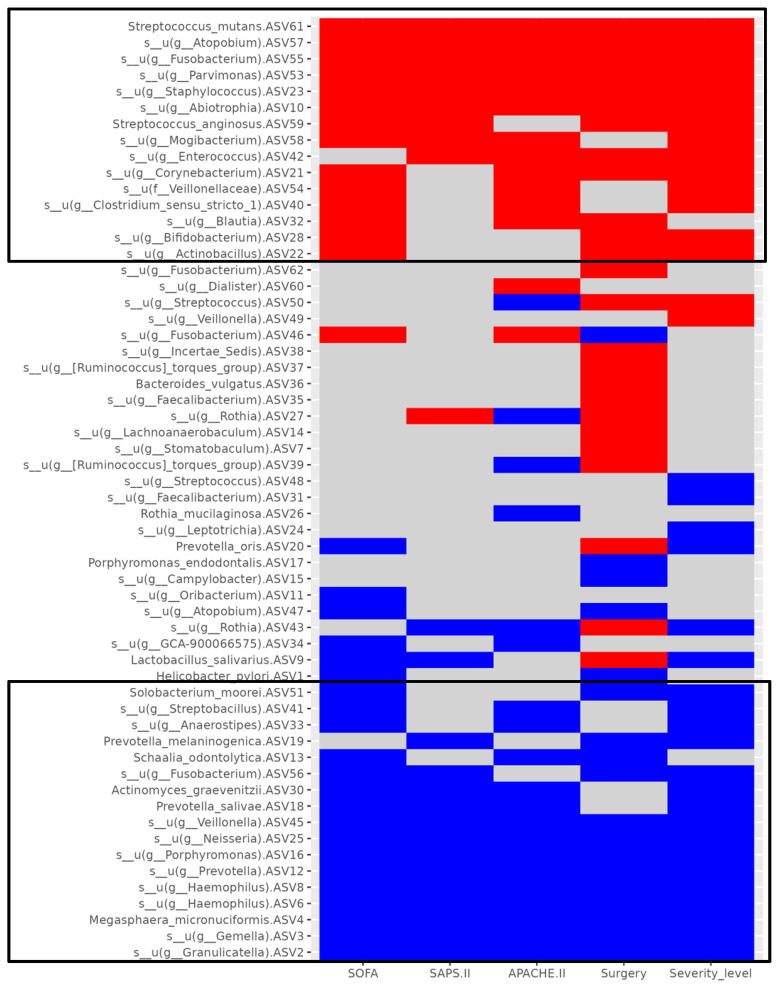
Bacteria associated with more severe (red) and milder (blue) courses of acute pancreatitis (AP) according to five indicators: SOFA, SAPS II, APACHE II scores, the need for surgical intervention (Surgery), and severity levels (mild, moderately severe, or severe). Black frames indicate groups of bacteria associated only positively or only negatively with at least 3 out of 5 indicators.

**Figure A2:**
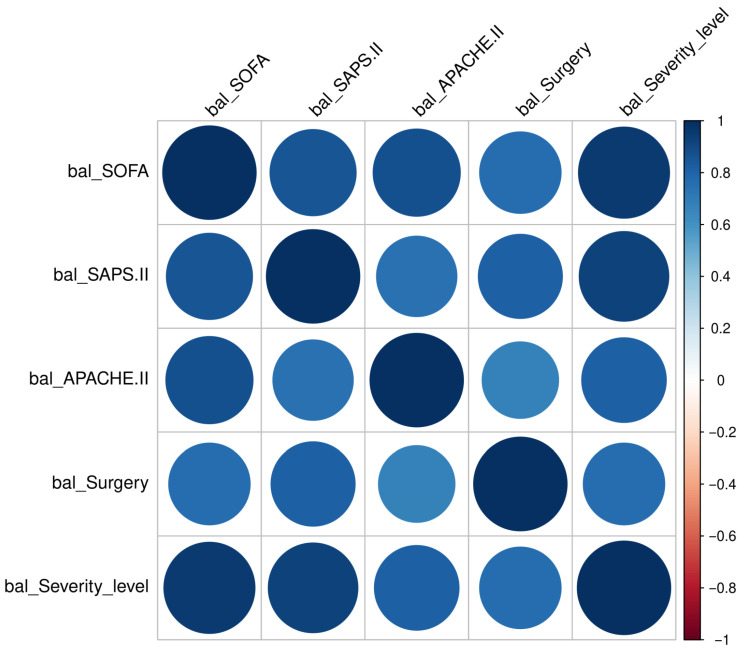
Spearman correlation of microbial signatures across various disease severity indicators, sample groups, and duration of pain syndrome. All correlations are significant (p.adj < 0.05). “Bal” denotes the balance value of groups of microbes associated with a particular indicator. “Surgery” denotes the surgical intervention due to purulent-septic complications. “Severity level”—severity of the acute pancreatitis (mild, moderately severe, or severe).

As the third step (Figure 2), we compared the results for all five severity measures. Notably, the microbial groups associated with more severe and milder forms of the disease overlapped across different severity metrics, and the balance values themselves were significantly correlated (p.adj < 0.1, Spearman correlation, Figure A1 and Figure A2). Based on this, we selected taxa that were consistently associated with multiple severity indicators and used the balance between these groups as a composite microbial marker—termed the PSMI.

**Figure 3:**
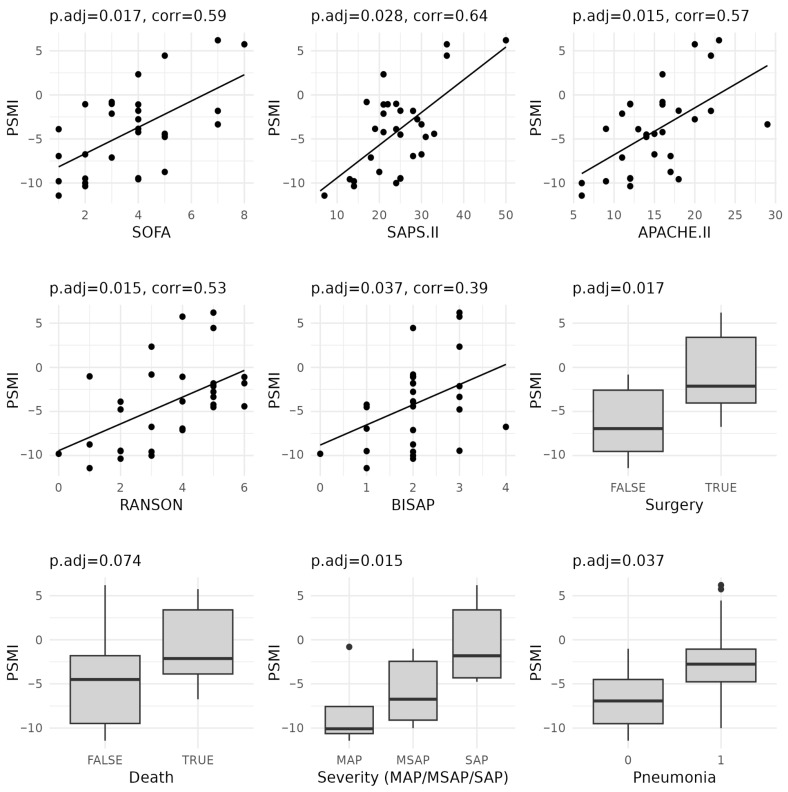

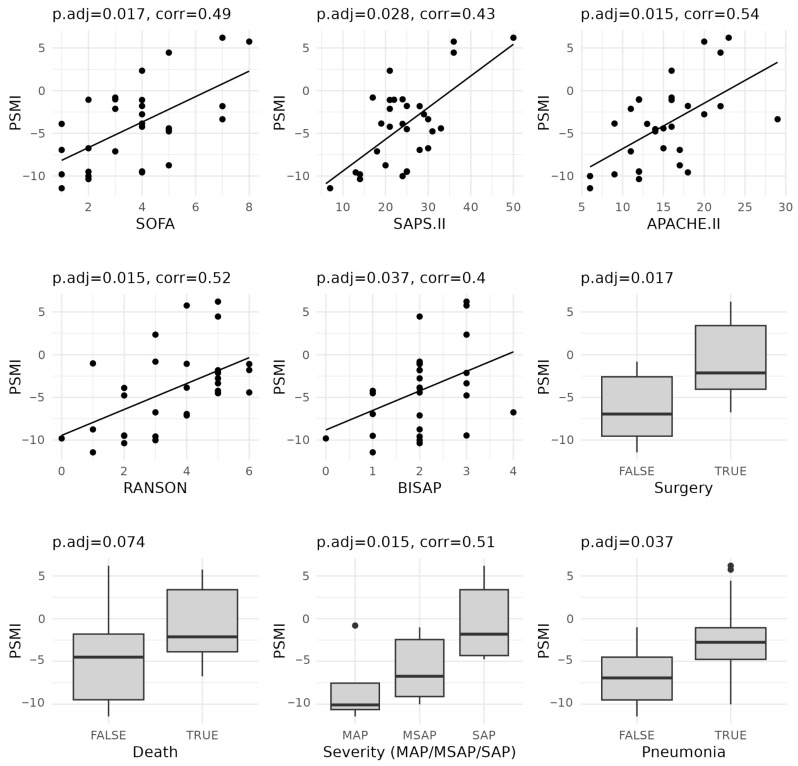
Relationship between pancreatitis severity microbial index (PSMI) values and clinical severity assessment factors (only those with p.adj < 0.1) and day of hospital admission relative to the onset of abdominal pain. MAP—mild acute pancreatitis, MSAP—moderately severe acute pancreatitis, SAP—severe acute pancreatitis. Statistical significance was estimated with Spearman’ correlation testing for continuous values (with severity treated as a numerical value) and Mann–Whitney test for categorical values.

The PSMI was significantly (*p* < 0.1, Table A3) associated with five out of seven severity scoring systems and all four outcome-related clinical parameters (Figure 3). The area under the receiver operating characteristic (ROC) curve (AUC) for predicting key outcomes based on the PSMI was 0.73 for mortality, 0.79 for complications requiring surgical intervention, 0.76 for pneumonia, and 0.78 for severe acute pancreatitis (SAP). A PSMI value below zero indicated that, on average, bacteria associated with severe disease were more prevalent than those linked to milder forms. However, even among patients with SAP or those who underwent surgery, the median PSMI values remained negative. This raised the question of identifying clinically meaningful threshold values of the PSMI indicative of adverse outcomes. An analysis of the relative abundances of taxa included in the PSMI (Figure 3) showed that microbes from the group associated with mild disease were consistently more abundant in patients with mild pancreatitis and less abundant in those with severe forms. Conversely, taxa associated with severe pancreatitis were found at lower levels in mild cases and increased in patients with severe pancreatitis.

#### 3.6.3. Microbiota Deterioration in the Absence of Treatment

**Figure 4:**
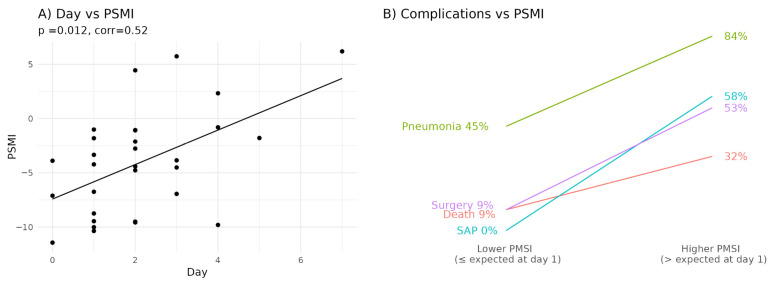
Deterioration of gut microbiota in the absence of specialized medical care in patients with acute pancreatitis and its prognostic implications. (**A**) Pancreatitis severity microbial index (PSMI) values in acute pancreatitis, showing a positive correlation with the number of days from symptom onset to hospital admission (Day). (**B**) Relationship between adverse outcomes of acute pancreatitis and the PSMI. SAP—severe acute pancreatitis. “Surgery” denotes the surgical intervention due to purulent-septic complications. SAP denotes severe acute pancreatitis.

We investigated the dynamics of the PSMI in relation to the time of hospital admission after the onset of abdominal pain. A statistically significant positive correlation was found between the PSMI and the time elapsed prior to admission (Figure 4A). This suggests that, without treatment, the proportion of microbes associated with adverse outcomes tends to increase as time progresses after symptom onset.

**Figure 5:**
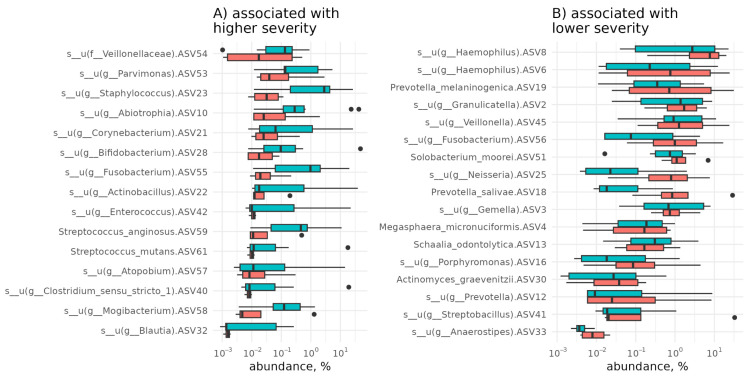
Relative abundance of microbes associated with severe (**A**) and mild (**B**) acute pancreatitis across patients with varying disease severity. Red denotes mild acute pancreatitis, and green indicates severe acute pancreatitis. ASV number corresponds to the identifier of the unique amplicon sequence variant.

Remarkably, based on linear regression analysis of the current data (Figure 4A), the average PSMI on the first day after symptom onset was approximately −5.8. Most patients with this or lower PSMI values survived, did not require surgical intervention, did not develop pneumonia, and were categorized as having mild (MAP) or moderately severe (MSAP) pancreatitis, rather than severe (SAP) forms (Figure 4B). In contrast, patients with higher PSMI values were significantly more likely to develop complications and experience a more severe disease course (Table A4). Although there was a trend toward worse clinical outcomes among patients admitted later (≥2 days after symptom onset) compared to those admitted within the first 24 h, this association did not reach statistical significance and warrants validation in larger cohorts (Figure 5B). Nonetheless, early admission (day 0–1) appears to be a favorable prognostic marker from the perspective of gut microbiota composition. Still, a more precise prediction of complications and disease severity is provided by the PSMI value itself.

## 4. Discussion

In this study, we found that the microbiota of the upper intestinal tract may serve as a predictor of acute pancreatitis (AP) severity. The relative abundance of specific bacterial taxa in small intestinal samples collected at the time of admission was correlated with disease severity, as evaluated post hoc using five out of seven international clinical scoring systems, as well as with key clinical outcomes, including the development of infected necrosis requiring surgical intervention, pneumonia, and either death or recovery. Additionally, alpha diversity was associated with one of the scoring systems. Based on these findings, we proposed a novel metric—the pancreatitis severity microbial index (PSMI)—which allows for the prediction of disease progression based on gut microbiota composition at the time of ICU admission.

The association between gut microbiota and the severity of AP has been described previously. There were reported alterations in the gut microbial community in patients with both chronic pancreatitis and AP [21,41], with their specific correlation with disease severity [12,42]. In a study by Brubaker et al. [42], the authors explored the use of stool microbiota composition to predict the development of AP, proposing a classifier for predicting the development of pancreatic necrosis based on fecal microbial profiles. The accuracy of their model (AUC) was comparable to that of our PSMI-based classifiers in predicting adverse outcomes and complications. However, to date, all available studies have focused on the microbiota of the large intestine, despite evidence suggesting that the main disruptions in AP occur in the small intestine. The microbial taxa captured by the PSMI differ notably from those identified in stool analyses as associated with the presence and severity of symptoms. For instance, while *Bifidobacterium* is typically reduced in stool samples from patients with severe AP, our findings show increased abundance of this genus in the small intestine of patients with more severe disease. Similarly, *Escherichia*/*Shigella*—often reported as linked to worse outcomes—was detected in only 3 out of 30 samples in our cohort. All three patients survived, with one requiring surgical intervention for severe AP, while the remaining two experienced moderately severe disease.

At present, the proposed PSMI index does not yet solve the problem of rapid prediction of disease progression, as the assessment of microbiota composition using 16S rRNA sequencing still takes too long. Potentially, this limitation could be overcome by developing a quantitative PCR panel to identify the specific microbes whose relative abundance determines the PSMI value. The development of such a system would require a study on a larger cohort and the use of sequencing methods capable of identifying microbiota composition at the species level. A larger sample size is necessary for the accurate evaluation of the index’s sensitivity and specificity. Species-level characterization is essential, as our study showed that different representatives of the same bacterial genus (in our case, *Fusobacterium*) may be associated with either milder or more severe disease forms. This can be completed by shotgun sequencing or full-length 16S rRNA gene sequencing. When developing PCR, it is also necessary to assess the possibility of reducing the number of microbial taxa used to calculate the PSMI, since in the current version, there are 32 of them.

The development of a PCR panel would reduce the PSMI calculation time to several hours and significantly lower diagnostic costs. However, even these few hours may be too long for urgent clinical decision-making. Furthermore, the sampling method remains relatively invasive, as it requires upper gastrointestinal endoscopy.

Nevertheless, the results of this study suggest that the state of the microbiota has significant prognostic value, which correlates with the time interval between the onset of pain and hospital admission with initiation of qualified medical care. Therefore, the duration of this interval may itself serve as a prognostic factor. Accordingly, the severity of AP can be initially assessed quickly and non-invasively based on the length of this interval and later refined using PCR diagnostics. Based on our findings, initiation of treatment within the first 24 h after the onset of pain appears to be critical. This finding should be confirmed in a larger cohort. If confirmed, it may serve as the basis for establishing new standards for the timely provision of medical care to patients with AP.

The main limitation of the present study is that it does not allow for specific conclusions regarding the mechanisms of interaction between the human body and the microbiota. This is due both to the relatively small sample size and to the method used for microbiota profiling: 16S rRNA sequencing does not provide accurate information on the metabolic activity of the microbial community. To address this, it is necessary to investigate the microbiota’s metabolic potential using shotgun metagenomic sequencing, transcriptomic profiling, or metabolomic analysis to evaluate actual microbial activity, along with clinical markers of host metabolism. Such an approach would help clarify whether changes in the microbiota are merely a consequence of organ dysfunction or if they may also contribute to the development of certain complications. These results could deepen our understanding of disease pathogenesis, facilitate the development of novel therapeutic strategies and preventive measures for complications in AP, and help assess the need for and methods of microbiota rehabilitation after patient recovery.

## 5. Conclusions

Although this study does not yet provide a ready-to-use tool for predicting the course of pancreatitis, it highlights the potential value of further investigation into the small intestinal microbiota. Future prognostic tools may be developed based on the assessment of microbial composition in the small intestine and the duration of abdominal pain prior to hospital admission. In addition, this research underscores the importance of exploring the interactions between the small intestinal microbiota and the human body, as well as identifying possible therapeutic interventions targeting these interactions.

## 6. Study Limitations

The main limitations of the study refer to the study design: the absence of a control group, dynamical observation, and small sample size.

The control group was not included in the study due to an ethical concern, namely, the invasiveness of the procedure of sample collection.

Dynamic assessment of small intestinal microbiota during treatment would require repeated sampling; however, logistical and ethical constraints prevent the acquisition of a complete picture of microbial changes in the small intestine. These limitations are primarily due to ethical concerns related to repeated gastroenteroscopy procedures and the financial burden of reintroducing a nasointestinal tube solely for research purposes. The analysis of the dynamic would also require a more uniform and larger sample, as the dynamics are affected by the treatment.

The process of obtaining small intestine lavage samples is highly dependent on the skill of the endoscopist and requires strict adherence to the standardized protocol. Failure to follow prescribed procedures can lead to substantial variability in results, both within the same operator and across different observers. This is why, at this point, we were not able to collect a large cohort.

Thus, this study’s design does not allow for describing differences between healthy and unhealthy small intestinal microbiomes, nor does it allow for estimating the interaction between the microbiome and the host’s health during the treatment. All the results concern only variability in the microbiome of untreated subjects. We would like to emphasize that the list of taxa included in PSMI is especially sensitive to the small sample size, as the human gut microbiome is known to be highly variable between subjects. The sample size makes the results sensitive to possible biases, such as self-treatment of patients before admission to the hospital or concomitant diseases that may also affect the small intestinal microbiome.

To develop targeted therapeutic strategies, further studies are needed to investigate the metabolic activity of the microbiota in patients with acute pancreatitis (AP) of varying severity. This includes analysis of bacterial metabolism, the influence of bacterial substrates on disease progression, and the relationship between microbial metabolism, clinical manifestations, and complications.

To further validate the proposed association between AP severity, alterations in small intestinal microbiota, and the frequency of septic complications, additional studies are required. These should include an analysis of bacterial peptides in various biological fluids from patients with AP.

## Figures and Tables

**Table 1:** Distribution of acute pancreatitis (AP) severity and frequency across age groups. MAP—mild acute pancreatitis, MSAP—moderately severe acute pancreatitis, SAP—severe acute pancreatitis.

AP Severity	Total	21–34 yrs	41–59 yrs	60–78 yrs
	*n* (%)			
Mild (MAP)	4 (13.3%)	3 (10%)	1 (3.3%)	0
Moderately severe (MSAP)	15 (50%)	3 (10%)	8 (26.7%)	4 (13.3%)
Severe (SAP)	11 (36.7%)	2 (6.7%)	7 (23.3%)	2 (6.7%)

**Table 2:** Etiological forms of acute pancreatitis and their gender distribution.

Etiology	Female	Male
	*n* (%)	
Idiopathic	1 (33.3%)	2 (66.7%)
Biliary	2 (40%)	3 (60%)
Alimentary (alcohol-related)	4 (18.2%)	18 (81.8%)

**Table 3:** Severity characteristics according to the clinical scoring systems.

Scale	Score (Median (Q1; Q3))
APACHE II	15.0 (12; 18)
SOFA	4.0 (2; 5)
BISAP	2.0 (2; 2.75)
SIRS	2.0 (2; 2.75)
SAPS II	24.0 (20.3; 28.8)
RANSON	4.0 (2; 5)
MARSHAL	4.0 (3; 5)

## Data Availability

The data presented in this study are available on request from the corresponding author. Our in-stitute’s policy does not permit the publication of such information in the public domain.

## Abbreviations

The following abbreviations are used in this manuscript:

AP	Acute pancreatitis
ASV	Amplicon sequence variant
ICU	Intensive care unit
MAP	Mild acute pancreatitis,
MSAP	Moderately severe acute pancreatitis
PSMI	Pancreatitis severity microbial index
SAP	severe acute pancreatitis
SI	Shock Index

## Appendix A

**Table A1:** Statistical significance of the association of the species composition of the microbiota with clinical data. Multiple testing adjustment was performed using the Benjamini–Hochberg method; *—Factors for which the association with beta diversity was significant before multiple testing adjustment (*p* < 0.1).

Parameter	Significance of Association with Aitchison Beta Diversity (PERMANOVA)			Significance of Association with Alpha Diversity (Spearman Correlation for Ordinal Scales and Mann–Whitney Test for Other Variables)		
	*p*	p.adj	Level of Significance (“-”—p.adj < 0.1)	*p*	p.adj	Level of Significance (“-”—p.adj < 0.1)
SOFA	0.072 *	0.158		0.0295	0.153	
SAPS.II	0.018 *	0.099	-	0.745	0.819	
APACHE.II	0.062 *	0.158		0.372	0.584	
RANSON	0.109	0.200		0.947	0.947	
MARSHALL	0.584	0.584		0.0754	0.207	
BISAP	0.332	0.365		0.249	0.456	
SIRS	0.253	0.309		0.0545	0.060	-
Surgery (Surgical intervention due to purulent-septic complications)	0.011 *	0.099	-	0.0417	0.153	
Death	0.251	0.309		0.532	0.732	
Severity (MAP/MSAP/SAP)	0.069 *	0.158		0.642	0.785	
Pneumonia	0.202	0.309		0.237	0.457	

**Table A2:** Statistical significance of the association of severity measures with the found balances (the balance taxa members are shown in Figure A1). Multiple testing adjustment was performed using the Benjamini–Hochberg method.

Parameter	Significance of Association with Corresponding Balance (Spearman Correlation for Ordinal Scales and Mann–Whitney Test for Other Variables)		
	*p*	p.adj	Level of Significance (**—p.adj < 0.01, ***—p.adj < 0.001, ****—p.adj < 0.00011)
SOFA	0.0002	0.0002	***
SAPS II	0.0047	0.00471	**
APACHE II	0.0000	0.0000	****
Surgical intervention due to purulent-septic complications	0.0000	0.0000	****
Severity (MAP/MSAP/SAP)	0.0001	0.0002	***

**Table A3:** Statistical significance of the association of pancreatitis severity microbial index (PSMI) with clinical data and the day of admission to the ICU from the onset of pain syndrome. Multiple testing adjustment was performed using the Benjamini–Hochberg method. MAP—mild acute pancreatitis, MSAP—moderately severe acute pancreatitis, SAP—severe acute pancreatitis.

Parameter	Significance of Association with Aitchison PSIM (Spearman Correlation for Ordinal Scales and Mann–Whitney Test for Other Variablespermanova)		
	*p*	p.adj	Level of Significance (*—p.adj < 0.05, “-”—p.andj < 0.1)
SOFA	0.01	0.02	*
SAPS II	0.02	0.03	*
APACHE II	0.00	0.01	*
RANSON	0.00	0.01	*
MARSHALL	0.79	0.79	
BISAP	0.03	0.04	*
SIRS	0.26	0.28	
Surgery (Surgical intervention due to purulent-septic complications)	0.01	0.02	*
Death	0.06	0.07	-
Severity (MAP/MSAP/SAP)	0.00	0.01	*
Pneumonia	0.03	0.04	*
Day of hospital admission from onset of pain syndrome	0.01	0.02	*

**Table A4:** Statistical significance of the association between late admission to the hospital and a high pancreatitis severity microbial index (PSMI) value with the incidence of complications. Multiple testing adjustment was performed using the Benjamini–Hochberg method.

Parameter	Long (>2 Days) Period From Onset of Pain Syndrome to Hospital Admission			High (Higher than Typical for 1 Day from the Onset of Pain Syndrome) PSMI Values		
	*p*	p.adj	Level of Significance (“-”—p.adj < 0.1)	*p*	p.adj	Level of Significance (*—p.adj < 0.05, “-”—p.adj < 0.1)
Surgery (Surgical intervention due to purulent-septic complications)	0.121	0.243		0.023	0.046	*
Death	0.669	0.669		0.215	0.215	
Pneumonia	0.102	0.243		0.042	0.056	-
Severe course of acute pancreatitis	0.442	0.59		0.002	0.006	*
